# Gaps in Public Awareness About BRCA and Genetic Testing in Prostate Cancer: Social Media Landscape Analysis

**DOI:** 10.2196/27063

**Published:** 2021-09-20

**Authors:** Stacy Loeb, Philip Massey, Amy E Leader, Sameer Thakker, Emily Falge, Sabina Taneja, Nataliya Byrne, Meredith Rose, Matthew Joy, Dawn Walter, Matthew S Katz, Risa L Wong, Preethi Selvan, Scott W Keith, Veda N Giri

**Affiliations:** 1 Department of Urology and Population Health New York University School of Medicine New York, NY United States; 2 Manhattan Veterans Affairs New York, NY United States; 3 Department of Community Health and Prevention, Dornsife School of Public Health, Drexel University Philadelphia, PA United States; 4 Department of Medical Oncology Sidney Kimmel Cancer Center Thomas Jefferson University Philadelphia, PA United States; 5 Department of Urology, New York University School of Medicine New York, NY United States; 6 Department of Radiation Medicine, Lowell General Hospital Lowell, MA United States; 7 Department of Medical Oncology, University of Washington Seattle, WA United States; 8 Division of Biostatistics, Department of Pharmacology and Experimental Therapeutics, Sidney Kimmel Medical College, Thomas Jefferson University Philadelphia, PA United States; 9 Department of Cancer Biology Thomas Jefferson University Philadelphia, PA United States; 10 Department of Urology Thomas Jefferson University Philadelphia, PA United States

**Keywords:** genetic testing, BRCA, prostate cancer, breast cancer, social media, infodemiology

## Abstract

**Background:**

Genetic testing, particularly for *BRCA1/2*, is increasingly important in prostate cancer (PCa) care, with impact on PCa management and hereditary cancer risk. However, the extent of public awareness and online discourse on social media is unknown, and presents opportunities to identify gaps and enhance population awareness and uptake of advances in PCa precision medicine.

**Objective:**

The objective of this study was to characterize activity and engagement across multiple social media platforms (Twitter, Facebook, and YouTube) regarding *BRCA* and genetic testing for PCa compared with breast cancer, which has a long history of public awareness, advocacy, and prominent social media presence.

**Methods:**

The Symplur Signals online analytics platform was used to obtain metrics for tweets about (1) #BRCA and #breastcancer, (2) #BRCA and #prostatecancer, (3) #genetictesting and #breastcancer, and (4) #genetictesting and #prostatecancer from 2016 to 2020. We examined the total number of tweets, users, and reach for each hashtag, and performed content analysis for a subset of tweets. Facebook and YouTube were queried using analogous search terms, and engagement metrics were calculated.

**Results:**

During a 5-year period, there were 10,005 tweets for #BRCA and #breastcancer, versus 1008 tweets about #BRCA and #prostatecancer. There were also more tweets about #genetictesting and #breastcancer (n=1748), compared with #genetic testing and #prostatecancer (n=328). Tweets about genetic testing (12,921,954) and *BRCA* (75,724,795) in breast cancer also had substantially greater reach than those about PCa (1,463,777 and 4,849,905, respectively). Facebook *groups* and *pages* regarding PCa and *BRCA*/genetic testing had fewer average members, new members, and new posts, as well as fewer likes and followers, compared with breast cancer. Facebook *videos* had more engagement than YouTube videos across both PCa and breast cancer content.

**Conclusions:**

There is substantially less social media engagement about *BRCA* and genetic testing in PCa compared with breast cancer. This landscape analysis provides insights into strategies for leveraging social media platforms to increase public awareness about PCa germline testing, including use of Facebook to share video content and Twitter for discussions with health professionals.

## Introduction

Genetic testing, particularly for *BRCA1* and *BRCA2*, has an increasing role in prostate cancer (PCa) management, screening, and hereditary cancer risk assessment [1-4]. Up to 12%-15% of metastatic disease and 5%-7% of early stage disease involve inherited genetic mutations in cancer risk genes [5,6]. PCa is the leading cancer diagnosed in US men, and inherited PCa impacts thousands of men [7]. Furthermore, hereditary cancer has important implications for family members, informing additional cancer risks and screening measures. Importantly, recommendations for PCa genetic testing have significantly expanded to include a large subset of men with or at risk for PCa [1,3,4]. For men with metastatic, castration-resistant PCa who carry *BRCA* mutations, the FDA has approved 2 poly-ADP ribose polymerase (PARP) inhibitors as targeted therapy after progression on standard therapy [8-10]. *BRCA* mutation status is also included in guidelines for PCa screening [4], and men with *BRCA2* mutations have more reclassification during active surveillance for favorable-risk disease [11].

Despite the importance of genetic factors in PCa management and hereditary cancer risk, the extent of public awareness is unclear. Previous studies have shown that public awareness and social media discourse are substantially greater for breast cancer compared with PCa [12,13]; however, these studies did not investigate discussions about genetics. Breast cancer is the leading cancer diagnosis among US women [7], and is a useful comparator for PCa because both can be inherited, and genetic mutations in *BRCA1/2* also affect screening and treatment recommendations in breast cancer [4,14].

As much as 3 in 4 US adults use 1 or more social media sites [15]. People increasingly use social media to look for health information, share their experiences, and communicate with others, which ultimately impacts their health beliefs and behaviors [16-18]. Social media provides unique insights into how people talk about, behave, and look for an array of health topics. These data have been used to inform prevention programming and messaging, and to scale-up prevention efforts and increase reach [19,20].

This topic is important, as recent data suggest that germline testing is underutilized in PCa [21], and that participating in social networks influences clinical decision making and health behaviors among patients with PCa [22]. From prevention, to treatment, to survivorship, social media provides an important space for communities and the general public to learn and share information about cancer and cancer prevention [20,23-25].

Our objective was to examine the current social media landscape regarding *BRCA* and genetic testing in PCa relative to breast cancer to provide insights into public awareness and inform strategies to enhance dissemination.

## Methods

We characterized activity and engagement across multiple social media platforms (Twitter, Facebook, and YouTube) regarding *BRCA* and #genetictesting for PCa compared with breast cancer.

### Twitter

The Symplur Signals platform was used to examine analytics for all tweets between 2016 and 2020 with the hashtags (1) #BRCA AND #breastcancer, (2) #BRCA AND #prostatecancer, (3) #genetictesting AND #breastcancer, and (4) #genetictesting AND #prostatecancer. We calculated the total number of tweets, users, and impressions (ie, potential accounts reached).

To further characterize the content and contributors, in June 2019, we exported all unique 2018 tweets for each hashtag and manually coded all PCa tweets due to the smaller sample size, a random 10% sample about #breastcancer #BRCA, and a random 50% sample about #breastcancer #genetictesting. A codebook was created through team consensus, based on our previous work [26]. Perceived race/ethnicity was coded by team consensus, as in prior studies [27]. Misinformation was assessed in comparison to guidelines and published literature [28]. The codebook was tested in a random sample with checks to verify intercoder variability and refined by the study team. Disagreements about codes were resolved by consensus.

### Facebook

From March to April 2020, we searched Facebook using the same 4 terms. To mitigate bias associated with Facebook’s user-centric search function, we cleared and unlinked prior account information [29]. The first 40 results for each term were examined. The Facebook search included the categories “Groups,” “Pages,” and “Videos.” We excluded duplicates and unrelated results.

We examined public metadata for Facebook groups, pages, and videos. For groups, we examined average number of members, average number of new posts and members within 30 days, and public versus private. For pages, we examined average followers, like counts, and date of page creation. For groups and pages, we analyzed their primary focus based on the provided descriptions, including awareness, support, treatment, research, and news (not mutually exclusive). For public Facebook videos, we counted average views, likes, and comments at the time of collection.

### YouTube

From March to April 2020, we searched YouTube using the same 4 terms as above after clearing account history, and examined the first 40 results for each. We excluded duplicates and unrelated results. We counted average views, likes, and comments at the time of collection. We standardized likes per video, views, and engagement rates.

### Statistical Analysis

Both PCa search terms were combined and compared with breast cancer terms. Summary statistics and 95% confidence intervals were calculated using SAS (SAS Institute) and Stata/IC 16 (StataCorp).

## Results

### Twitter

**Figure 1:**
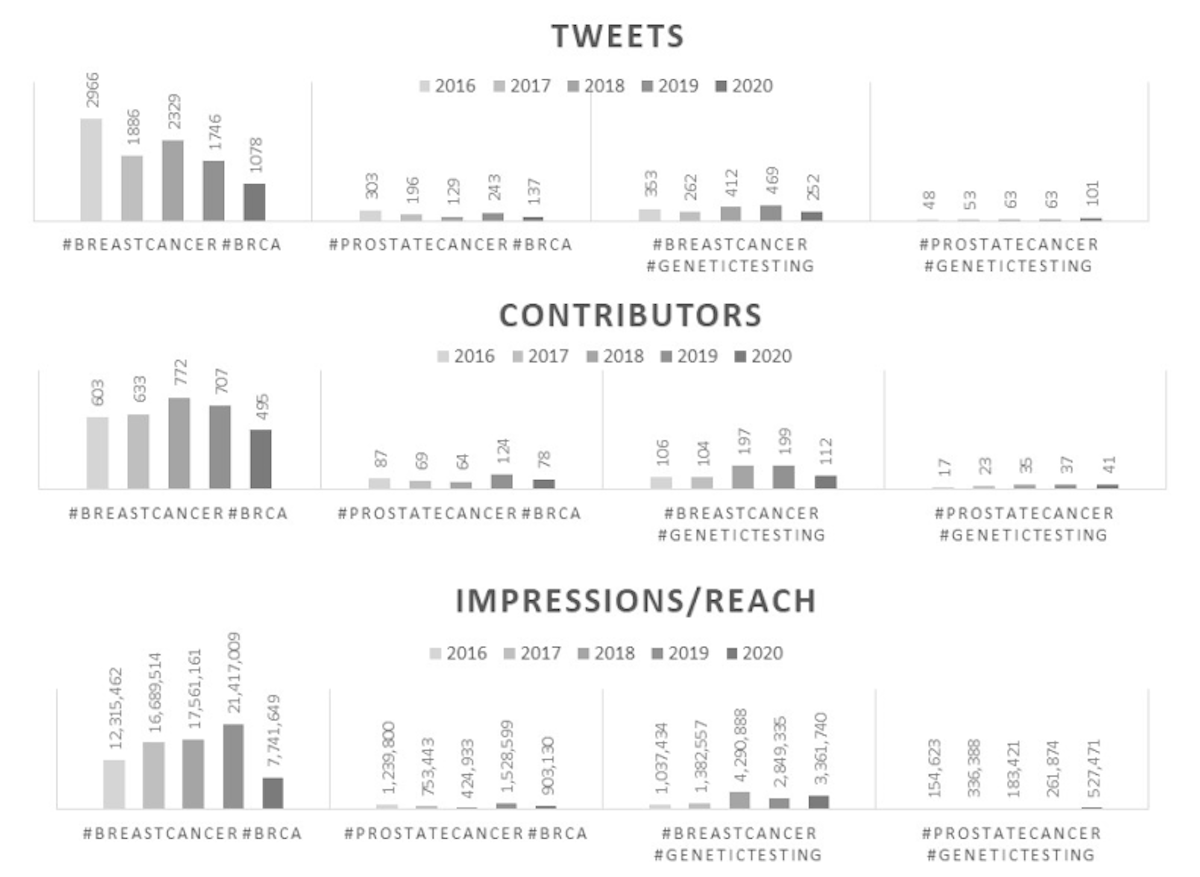
Comparison of Tweets, Contributors, and Impressions/Reach for BRCA and Genetic Testing in Breast Cancer versus Prostate Cancer (2016-2020).

From 2016 to 2020, in PCa and breast cancer there were 1008 and 10,005 tweets about *BRCA*, and 328 and 1748 tweets about genetic testing, respectively (see Figure 1 for trend over time). Users and reach were also substantially higher for *BRCA* and genetic testing in breast cancer relative to PCa throughout 5-year period.

Coding of a subset of Tweets is shown in Multimedia Appendix 1. The most common type of post was sharing an article link. Sentiment was mostly neutral. Misinformation was rare. Gender was mentioned more often than race. For tweets about *BRCA*, the most common tweeters were foundations/advocacy groups followed by health professionals, whereas for tweets about genetic testing, foundations/advocacy groups and commercial entities were the most common. Among individual Twitter contributors, most were perceived as White and female for all topics except PCa genetic testing for which the largest number of users were perceived as White males.

### Facebook Groups and Pages

Table 1 shows results for 73 Facebook groups and 80 Facebook pages. PCa groups had fewer average members than breast cancer groups. This was consistent with other variables including average number of posts and members in the past 30 days. For Facebook pages, breast cancer pages had higher followers and likes than PCa. Among PCa and breast cancer groups and pages, most focused on awareness, support, and treatment (Multimedia Appendix 2).

**Table 1:** Analysis of Facebook groups and pages comparing prostate and breast cancer in March 2020 (n=153).

Facebook feature		Prostate cancer *BRCA*/genetic testing^a^	Breast cancer *BRCA*/genetic testing^a^
**Groups (n=73)**			
	Total posts	35	38
	Average members (range)	1744 (36-13,767)	4203 (58-16,863)
	Average posts in the past 30 days (range)	198 (0-2816)	451 (3-2777)
	Average new members in the past 30 days (range)	61 (0-527)	117 (0-992)
	Public, n/N (%)	5/35 (14)	6/38 (16)
	Created range	2006-2019	2007-2019
**Pages (n=80)**			
	Total posts	40	40
	Average followers (range)	17,215 (13-225,550)	151,858 (6-5,079,917)
	Average likes (range)	17,422 (13-231,855)	174,785 (6-5,989,522)
	Created range	2008-2019	2007-2019

^a^First 40 search results included from each category; Facebook groups/pages within categories are mutually exclusive.

### Facebook and YouTube Videos

Among 230 videos analyzed (Table 2), Facebook videos exhibited higher view counts and more comments. Average likes per view for PCa videos on Facebook were similar to that of YouTube, while breast cancer had more likes per view on Facebook than YouTube. Engagement rate for PCa was slightly higher on YouTube, whereas for breast cancer it was higher on Facebook.

**Table 2:** Comparing Facebook and YouTube video characteristics by means and 95% confidence intervals, March-April 2020.

Characteristics	Prostate cancer *BRCA*/genetic testing (n=119)			Breast cancer *BRCA*/genetic testing (n=111)	
	Facebook (n=58)	YouTube (n=61)	Facebook (n=66)		YouTube (n=45)
Views	22,595 (4530-40,659)	282 (203-360)	22,169 (4529-40,162)		3,250 (1467-5033)
Likes	204 (42-366)	3 (2-4)	227 (75-379)		13 (7-19)
Comments	29.4 (9.9-49.0)	0.2 (0.1-0.3)	17.8 (5.4-30.3)		0.5 (0.2-0.8)
Likes per view	0.018 (0.011-0.024)	0.012 (0.009-0.015)	0.026 (0.013-0.039)		0.005 (0.004-0.007)
Engagement rate^a^	1.90 (1.29-2.51)	1.31 (0.94-1.68)	2.77 (1.47-4.07)		0.58 (0.43-0.72)
Video length, mm:ss	6:28 (2:33-10:23)	10:14 (6:42-13:47)	3:17 (2:25-4:10)		7:16 (3:23-11:09)

^a^Engagement rate is the addition of number of likes, dislikes, and comments divided by the total number of views multiplied by 100. Videos within are mutually exclusive.

## Discussion

Our results show substantial discussion about *BRCA* and genetic testing on popular social networks, although with more participation and engagement for breast cancer than PCa. This corroborates previous studies showing that PCa in general has less social media engagement [12,13], and raises less funding through crowdfunding than breast cancer [30]. Since PCa germline testing guidelines have expanded [3,4], thousands of men are eligible for testing, which may inform management and hereditary cancer risk. As the majority of adults look online for health information and social media use among older adults continues to rise [15,31], a lack of social discourse about PCa and genetic testing may hinder men from knowing that this option is available.

Our results raise concern for modest social media activity and participation, and lack of public awareness about the importance of PCa germline testing; follow-up studies are needed to determine whether this is hindering the impact of genetic advances. Future research is also warranted to draw from the greater social media experience in breast cancer, and to examine the potential for targeted social media campaigns to increase awareness and uptake of genetic evaluation for PCa.

A positive finding of the study was the minimal presence of misinformation on Twitter related to genetic testing and *BRCA* in breast cancer or PCa. This is in stark contrast to previous studies suggesting a substantial amount of misinformation about PCa on other social networks such as YouTube [28]. A possible explanation was the high proportion of tweets from health care professionals and foundations.

A limitation of this study is that only a subset of social media platforms and posts including these specific hashtags/search terms was evaluated. Additionally, coding of certain variables, such as perceived gender and race/ethnicity, is inherently subjective. Strengths include the first landscape analysis of social media activity related to *BRCA* and genetic testing in PCa, compared with breast cancer. These data are useful to inform public awareness strategies. Although YouTube is the largest overall video-sharing network, we found that videos about genetic testing had greater reach on Facebook, suggesting that it should be used to disseminate video content to the public. In addition, we found that Twitter is a valuable resource to follow research updates about germline testing for health care professionals and other stakeholders.

In conclusion, there is substantially less social media activity regarding *BRCA* and genetic testing in PCa relative to breast cancer. These results highlight a major need to increase public awareness and support for genetic testing for PCa to enhance the impact of the precision medicine era.

## Appendix

Multimedia Appendix 1 Supplemental Table 1.

Multimedia Appendix 2 Supplemental Table 2.

## References

[1] Giri Veda N, Knudsen K, Kelly W, Cheng H, Cooney K, Cookson M, Dahut William, Weissman Scott, Soule Howard R, Petrylak Daniel P, Dicker Adam P, AlDubayan Saud H, Toland Amanda E, Pritchard Colin C, Pettaway Curtis A, Daly Mary B, Mohler James L, Parsons J Kellogg, Carroll Peter R, Pilarski Robert, Blanco Amie, Woodson Ashley, Rahm Alanna, Taplin Mary-Ellen, Polascik Thomas J, Helfand Brian T, Hyatt Colette, Morgans Alicia K, Feng Felix, Mullane Michael, Powers Jacqueline, Concepcion Raoul, Lin Daniel W, Wender Richard, Mark James Ryan, Costello Anthony, Burnett Arthur L, Sartor Oliver, Isaacs William B, Xu Jianfeng, Weitzel Jeffrey, Andriole Gerald L, Beltran Himisha, Briganti Alberto, Byrne Lindsey, Calvaresi Anne, Chandrasekar Thenappan, Chen David Y T, Den Robert B, Dobi Albert, Crawford E David, Eastham James, Eggener Scott, Freedman Matthew L, Garnick Marc, Gomella Patrick T, Handley Nathan, Hurwitz Mark D, Izes Joseph, Karnes R Jeffrey, Lallas Costas, Languino Lucia, Loeb Stacy, Lopez Ana Maria, Loughlin Kevin R, Lu-Yao Grace, Malkowicz S Bruce, Mann Mark, Mille Patrick, Miner Martin M, Morgan Todd, Moreno Jose, Mucci Lorelei, Myers Ronald E, Nielsen Sarah M, O'Neil Brock, Pinover Wayne, Pinto Peter, Poage Wendy, Raj Ganesh V, Rebbeck Timothy R, Ryan Charles, Sandler Howard, Schiewer Matthew, Scott E Michael D, Szymaniak Brittany, Tester William, Trabulsi Edouard J, Vapiwala Neha, Yu Evan Y, Zeigler-Johnson Charnita, Gomella Leonard G (2020). Implementation of Germline Testing for Prostate Cancer: Philadelphia Prostate Cancer Consensus Conference 2019. J Clin Oncol.

[2] Cheng Heather H, Sokolova A, Schaeffer E, Small E, Higano C (2019). Germline and Somatic Mutations in Prostate Cancer for the Clinician. J Natl Compr Canc Netw.

[3] National Comprehensive Cancer Network Clinical Practice Guidelines in Oncology (2021). Prostate Cancer Version 2.

[4] (2021). National Comprehensive Cancer Network Clinical Guidelines in Oncology (NCCN Guidelines®): Genetic/Familial High-Risk Assessment: Breast, Ovarian, and Pancreatic (Version 2.2021).

[5] Genetics of Prostate Cancer (PDQ®): Health Professional Version.

[6] Pritchard CC, Mateo J, Walsh MF, De Sarkar N, Abida W, Beltran H, Garofalo A, Gulati R, Carreira S, Eeles R, Elemento O, Rubin MA, Robinson D, Lonigro R, Hussain M, Chinnaiyan A, Vinson J, Filipenko J, Garraway L, Taplin M, AlDubayan S, Han GC, Beightol M, Morrissey C, Nghiem B, Cheng HH, Montgomery B, Walsh T, Casadei S, Berger M, Zhang L, Zehir A, Vijai J, Scher HI, Sawyers C, Schultz N, Kantoff PW, Solit D, Robson M, Van Allen EM, Offit K, de Bono J, Nelson PS (2016). Inherited DNA-Repair Gene Mutations in Men with Metastatic Prostate Cancer. N Engl J Med.

[7] American Cancer Society Cancer Facts and Figures 2021.

[8] Abida W, Patnaik A, Campbell D, Shapiro J, Bryce AH, McDermott R, Sautois B, Vogelzang NJ, Bambury RM, Voog E, Zhang J, Piulats JM, Ryan CJ, Merseburger AS, Daugaard G, Heidenreich A, Fizazi K, Higano CS, Krieger LE, Sternberg CN, Watkins SP, Despain D, Simmons AD, Loehr A, Dowson M, Golsorkhi T, Chowdhury S (2020). Rucaparib in Men With Metastatic Castration-Resistant Prostate Cancer Harboring a BRCA1 or BRCA2 Gene Alteration. JCO.

[9] de Bono J, Mateo J, Fizazi K, Saad F, Shore N, Sandhu S, Chi KN, Sartor O, Agarwal N, Olmos D, Thiery-Vuillemin A, Twardowski P, Mehra N, Goessl C, Kang J, Burgents J, Wu W, Kohlmann A, Adelman CA, Hussain M (2020). Olaparib for Metastatic Castration-Resistant Prostate Cancer. N Engl J Med.

[10] National Cancer Institute With Two FDA Approvals, Prostate Cancer Treatment Enters the PARP Era. National Cancer Instutute Cancer Currents Blog.

[11] Carter HB, Helfand B, Mamawala M, Wu Y, Landis P, Yu H, Wiley K, Na R, Shi Z, Petkewicz J, Shah S, Fantus RJ, Novakovic K, Brendler CB, Zheng SL, Isaacs WB, Xu J (2019). Germline Mutations in ATM and BRCA1/2 Are Associated with Grade Reclassification in Men on Active Surveillance for Prostate Cancer. Eur Urol.

[12] Loeb S, Stork B, Gold HT, Stout NK, Makarov DV, Weight CJ, Borgmann H (2017). Tweet this: how advocacy for breast and prostate cancers stacks up on social media. BJU Int.

[13] Vraga EK, Stefanidis A, Lamprianidis G, Croitoru A, Crooks AT, Delamater PL, Pfoser Dieter, Radzikowski JR, Jacobsen KH (2018). Cancer and Social Media: A Comparison of Traffic about Breast Cancer, Prostate Cancer, and Other Reproductive Cancers on Twitter and Instagram. J Health Commun.

[14] National Comprehensive Cancer Network Breast Cancer. Clinical Practice Guidelines in Oncology.

[15] Pew Research Center Social Media Fact Sheet.

[16] Korda H, Itani Z (2013). Harnessing social media for health promotion and behavior change. Health Promot Pract.

[17] Laranjo Liliana, Arguel A, Neves A, Gallagher A, Kaplan R, Mortimer N, Mendes Guilherme A, Lau Annie Y S (2015). The influence of social networking sites on health behavior change: a systematic review and meta-analysis. J Am Med Inform Assoc.

[18] Oh HJ, Lauckner C, Boehmer J, Fewins-Bliss R, Li K (2013). Facebooking for health: An examination into the solicitation and effects of health-related social support on social networking sites. Computers in Human Behavior.

[19] Eysenbach G (2011). Infodemiology and infoveillance tracking online health information and cyberbehavior for public health. Am J Prev Med.

[20] Prochaska JJ, Coughlin SS, Lyons EJ (2017). Social Media and Mobile Technology for Cancer Prevention and Treatment. Am Soc Clin Oncol Educ Book.

[21] Loeb S, Byrne N, Walter D, Makarov DV, Wise DR, Becker D, Giri VN (2020). Knowledge and practice regarding prostate cancer germline testing among urologists: Gaps to address for optimal implementation. Cancer Treat Res Commun.

[22] Huber J, Maatz P, Muck T, Keck B, Friederich H, Herzog W, Ihrig A (2017). The effect of an online support group on patients׳ treatment decisions for localized prostate cancer: An online survey. Urol Oncol.

[23] Himelboim I, Han JY (2014). Cancer talk on twitter: community structure and information sources in breast and prostate cancer social networks. J Health Commun.

[24] Chung JE (2014). Social networking in online support groups for health: how online social networking benefits patients. J Health Commun.

[25] Falisi AL, Wiseman KP, Gaysynsky A, Scheideler JK, Ramin DA, Chou WS (2017). Social media for breast cancer survivors: a literature review. J Cancer Surviv.

[26] Loeb S, Byrne NK, Thakker S, Walter D, Katz MS (2020). #ILookLikeAUrologist: Using Twitter to Discuss Diversity and Inclusion in Urology. Eur Urol Focus.

[27] Borno HT, Zhang S, Bakke B, Bell A, Zuniga KB, Li P, Chao K, Sabol A, Killeen T, Hong H, Walter D, Loeb S (2020). Racial disparities and online health information: YouTube and prostate cancer clinical trials. BJU Int.

[28] Loeb S, Sengupta S, Butaney M, Macaluso JN, Czarniecki SW, Robbins R, Braithwaite RS, Gao L, Byrne N, Walter D, Langford A (2019). Dissemination of Misinformative and Biased Information about Prostate Cancer on YouTube. Eur Urol.

[29] Intro to Facebook Search. Facebook.

[30] Loeb S, Taneja S, Walter D, Zweifach S, Byrne N (2018). Crowdfunding for prostate cancer and breast cancer. BJU Int.

[31] Pew Internet & American Life Project Health Online 2013.

