# Supplementary material for: Gaps in Public Awareness About BRCA and Genetic Testing in Prostate Cancer: Social Media Landscape Analysis
Source: JMIR Cancer. 2021 Sep 20;7(3):e27063. doi: 10.2196/27063 (PMC8550715; doi:10.2196/27063)
Supplement: Multimedia Appendix 1 [file cancer_v7i3e27063_app1.docx]

Supplemental Table 1. Manual analysis for subset of 2018 Tweets.

| **Characteristics** | ***BRCA*-**  **Breast cancer**  **n (%)** | ***BRCA*- prostate cancer**  **n (%)** | **Genetic testing- breast cancer**  **n (%)** | **Genetic testing- prostate cancer**  **n (%)** | **Total**  **n (%)** |
| --- | --- | --- | --- | --- | --- |
| Primary type of content  Link to article  Awareness messaging  Personal narrative  Social media promotion  Advertisement  Other | 211 (59)  32 (9)  38 (11)  26 (7)  10 (3)  40 (11) | 110 (54)  44 (21)  6 (3)  5 (2)  3 (1)  37 (18) | 129 (61)  24 (11)  7 (3)  5 (2)  22 (10)  24 (11) | 76 (85)  3 (3)  1 (1)  2 (2)  1 (1)  6 (7) | 526 (61)  103 (12)  52 (6)  38 (4)  36 (4)  107 (12) |
| Sentiment  Positive  Negative  Neutral/NA | 20 (6)  2 (1)  335 (94) | 29 (14)  3 (1)  173 (84) | 135 (64)  5 (2)  71 (34) | 26 (29)  2 (2)  61 (69) | 210 (24)  12 (1)  640 (74) |
| Discussion of Race/Ethnicity  Yes  No  Unknown/NA | 12 (3)  273 (76)  72 (20) | 7 (3)  198 (97)  0 | 7 (3)  204 (97)  0 | 0  89 (100)  0 | 26 (3)  764 (89)  72 (8) |
| Discussion of Gender  Yes  No  Unknown/NA | 52 (15)  246 (69)  59 (17) | 73 (36)  132 (64)  0 | 49 (23)  162 (77)  0 | 4 (4)  85 (96)  0 | 178 (21)  625 (73)  59 (7) |
| Type of Cancer Discussed  Breast only  Prostate only  Multiple cancers  Not specified | 308 (86)  1 (<1)  35 (10)  13 (4) | 1 (<1)  116 (57)  86 (42)  2 (1) | 192 (91)  0  15 (7)  4 (2) | 1 (1)  85 (96)  3 (3)  0 | 502 (58)  202 (23)  139 (16)  19 (2) |
| Type of Tweeter  Healthcare provider/scientist  Foundation/government org  Commercial entity  News/media/journal  Hospital/clinic  Blogger/wellness channel  Patient/Caregiver  Other/unknown | 69 (19)  59 (17)  44 (12)  36 (10)  27 (8)  41 (11)  36 (10)  45 (13) | 79 (39)  58 (28)  16 (8)  19 (9)  1 (<1)  8 (4)  18 (9)  6 (3) | 43 (20)  46 (22)  34 (16)  21 (10)  36 (17)  9 (4)  5 (2)  17 (8) | 21 (24)  19 (21)  13 (15)  17 (19)  7 (8)  9 (10)  1 (1)  2 (2) | 212 (25)  182 (21)  107 (12)  93 (11)  71 (8)  67 (8)  60 (7)  70 (8) |
| Perceived Gender of Tweeter  Female  Male  Cannot discern/NA | 100 (28)  47 (13)  210 (59) | 74 (36)  41 (20)  90 (44) | 29 (14)  22 (10)  160 (76) | 6 (7)  11 (12)  72 (81) | 209 (24)  121 (14)  532 (62) |
| Perceived Race/Ethnicity of Tweeter  White  Asian  Hispanic/LatinX  Black  Cannot Discern/Other/NA | 108 (30)  10 (3)  3 (1)  3 (1)  233 (65) | 96 (47)  7 (3)  4 (2)  5 (2)  93 (45) | 31 (15)  8 (4)  4 (2)  2 (1)  166 (79) | 11 (12)  4 (4)  2 (2)  0  72 (81) | 246 (29)  29 (3)  13 (2)  10 (1)  564 (65) |
| Continent of Tweeter  North America  Europe  Asia  Australia/Oceania  Africa  South America  Not specified | 190 (53)  45 (13)  12 (3)  6 (2)  0  2 (1)  102 (29) | 111 (54)  22 (11)  1 (<1)  3 (1)  1 (<1)  3 (1)  64 (31) | 141 (67)  8 (4)  12 (6)  4 (2)  5 (2)  0  41 (19) | 66 (74)  4 (4)  2 (2)  0  1 (1)  1 (1)  15 (17) | 508 (59)  79 (9)  27 (3)  13 (2)  7 (1)  6 (1)  222 (26) |
