# Supplementary material for: Gaps in Public Awareness About BRCA and Genetic Testing in Prostate Cancer: Social Media Landscape Analysis
Source: JMIR Cancer. 2021 Sep 20;7(3):e27063. doi: 10.2196/27063 (PMC8550715; doi:10.2196/27063)
Supplement: Multimedia Appendix 2 [file cancer_v7i3e27063_app2.docx]

Supplemental Table 2. Focus of Groups and Pages about BRCA/Genetic Testing in Prostate Cancer and Breast Cancer.

| **Groups and Pages Focus^b^ (n=153)** | **Prostate Cancer *BRCA*/ Genetic Testing^a^**  **n=73** | **Breast Cancer *BRCA*/Genetic Testing^a^**  **n=80** |
| --- | --- | --- |
| Awareness | 53 (72.6%) | 56 (70%) |
| Support | 49 (67.1%) | 59 (73.8%) |
| Treatment | 15 (20.5%) | 8 (10%) |
| News | 1 (1.4%) | 0 (0%) |
| Research | 0 (0%) | 0 (0%) |
| *a First 40 search results included from each category; Facebook groups/pages within categories are mutually exclusive.*  *b Group & page focus categories are not mutually exclusive.* | | |
